# Blind information reconciliation with variable step sizes for quantum key distribution

**DOI:** 10.1038/s41598-019-56637-y

**Published:** 2020-01-13

**Authors:** Zhihong Liu, Zhihao Wu, Anqi Huang

**Affiliations:** 1 College of Intelligence and Technology, National University of Defense Technology, Changsha, 410073 People’s Republic of China; 2 Institute for Quantum Information & State Key Laboratory of High Performance Computing, College of Computer, National University of Defense Technology, Changsha, 410073 People’s Republic of China

**Keywords:** Optics and photonics, Applied optics, Optical techniques, Electrical and electronic engineering

## Abstract

Quantum key distribution (QKD) generates symmetric keys between two authenticated parties with the guarantee of information-theoretically security. A vital step in QKD to obtain fully-matched key between two parties is information reconciliation. The blind reconciliation protocol provides a useful tool that corrects the mismatch in a wide range of qubit error rate (QBER) but without a prior error estimation. However, there is a contradiction between the reconciliation efficiency and the processing time in this protocol. In this work, we propose a blind reconciliation protocol with variable step sizes to relieve this contradiction. The analysis and simulation results show that the improved protocol inherits all the advantages of the original blind reconciliation protocol and can obtain better reconciliation efficiency with less operation time. The improved blind reconciliation protocol enhances the final secret key rate and accelerates the processing speed of a QKD system.

## Introduction

Quantum key distribution (QKD)^1,2^ allows two parties, usually called Alice and Bob, to share a pair of secret key via an insecure channel. QKD is proved to be information-theoretically secure owing to the solid foundation of quantum physics without computational assumptions^3–5^. The technology of QKD has been developed quickly in the past three decades and has achieved remarkable milestones. For example, QKD products has been commercialized with growing market^6^; QKD networks has been constructed in several countries around the world^7–9^; a QKD satellite realizes secure communication in global scale^10,11^. Thus, QKD is one of the most mature fields in quantum information.

In the implementation of QKD, a system operates two main phases to establish the shared key – the phase of quantum raw key exchange and the phase of classical post-processing^12^. At the first phase, Alice and Bob share the raw key by transmitting quantum states prepared by Alice through a quantum channel and measuring them by Bob. Then, sifting as the first step of classical post-processing helps Alice and Bob to maintain the cases that they are using the matched bases. The raw key is obtained after this step. However, the raw key shared between Alice and Bob may contain errors due to the channel noise and adversary’s attacks. To eliminate the mismatch of the raw key between Alice and Bob, the system runs information reconciliation to guarantee that the same string of key shared at both sides. The reconciled key maybe partially correlated with an adversary, since the adversary can interact with the quantum states during the raw key exchange via quantum channel and listen to the public information during the information reconciliation via the classical channel. Thus, privacy amplification is applied to remove the leaked information, thereby obtaining the final secret key share between Alice and Bob. The sifting, information reconciliation, and privacy amplification are called as the post-processing phase.

In the post-processing, it is obvious that information reconciliation is a vital step to eliminate the mismatch, which is also called error bits, in the raw key to guarantee that Alice and Bob share the same key^13^. Regarding information reconciliation, a commonly used method is Cascade protocol, which identifies the position of errors via dichotomizing search in each block^14^. Although Cascade protocol is simple to be implemented with relative high reconciliation efficiency, this protocol requires many rounds of communication between Alice and Bob. Thus, this information reconciliation protocol is time-consuming and occupies significant amount of communication resources in a QKD system. To reduce the rounds of communication, Winnow protocol is proposed, which uses Hamming code instead of a simple parity check to identify and correct errors^15^. However, Hamming code only can correct 1-bit error in each block, and its efficiency is much lower than the Shannon limit.

Low-density parity-check (LDPC) code^16,17^ is proposed to be used for the information reconciliation in a QKD system to reduce the rounds of communication and achieve high reconciliation efficiency^18,19^. Since LDPC code is able to correct multiple-bit errors, its reconciliation efficiency is close to the Shannon limit. Furthermore, only one round of communication is necessary to transmit the syndrome. Hence, the party (e.g. Alice or Bob) who receives the syndrome can use it to correct the errors. In practice, quantum channel is varying over time, and thus quantum bit error rate (QBER) is also changing^20^. In order to adapt to the different QBER, two techniques, puncturing^21,22^ and shortening^23^, are applied to the LDPC code to correct errors in one round of communication. This is called rate-adaptive information reconciliation^19,20,24^.

Based on the rate-adaptive information reconciliation, blind information reconciliation protocol is suitable to the situation that the QBER is unknown, in which the reconciliation procedure is processed *blindly* without information of QBER^25–28^. Because of no information about the QBER, the blind reconciliation protocol allows multiple rounds of communication to try to correct the errors by assuming different error rate in each round. The specific procedure of the blind reconciliation protocol is as follows. In the first round of communication, Alice assumes the minimal error rate and only transmits the syndrome to Bob, and all the auxiliary bits are punctured. If the reconciliation fails, Alice reveals a small amount of punctured bits, turning them to be shortened bits, which helps Bob to correct errors. With more rounds the protocol operates, more shortened bits are known by Bob. Until Bob successfully corrects all errors or the maximum of rounds reaches, the protocol ends. This protocol does not need the error estimation as prior information and can achieve high average efficiency^26^.

However, there is a trade off between the reconciliation efficiency and the time consumption of this protocol. On the one hand, the more rounds of interactivity allowed between Alice and Bob, the higher reconciliation efficiency can achieve. On the other hand, more rounds of interactivity take more time to complete the protocol. Practically, in a QKD system, the time consumption of the post-processing significantly affects the secret key rate. If the post-processing consumes much more time than the raw key exchange phase, the secret key rate will be limited by the speed of post-processing and thus cannot obtain high key rate.

To relieve the contradiction between the reconciliation efficiency and the time consumption, we propose a blind information reconciliation protocol with variable step sizes. Different from the original protocol, the proposed protocol gradually increases the amount of the shortened bits revealed in each round, rather than reveals constant amount of the shortened bits in each round. In this way, the proposed protocol can achieve better reconciliation efficiency while incurring similar or even less time consumption to complete the error correction. More specifically, the simulation results show that the proposed protocol can achieve improvement of the reconciliation efficiency while consuming less iteration time. Besides, by using a decoy-state BB84 QKD system, we demonstrate that the proposed protocol can improve the secret key rate. This is useful in practice when the QBER is not known in advance. In short, this protocol inherits all the advantages of the original blind information reconciliation protocol and can provide better reconciliation efficiency with less operation time than the original one.

The paper is organized as follows. In Sec. 2, we introduce the protocol of blind information reconciliation with variable step sizes. The corresponding simulation results that compared to the original blind information reconciliation protocol are shown in Sec. 3. We further analysis the effect of the improved reconciliation efficiency owing to the variable step sizes on the secret key rate of QKD in Sec. 4. The conclusion is drawn in Sec. 5.

## Blind Reconciliation With Variable Step Sizes

Blind reconciliation can cover a wide range of QBER by using only one LDPC code. This is due to the technique of rate-adaptive reconciliation. Thus, in this section, we first describe the working principle of rate-adaptive information reconciliation. Based on this, we introduce the protocol of blind reconciliation with variable step sizes that we propose.

### Rate-adaptive information reconciliation

For a given LDPC code $C(n,k)$, there are $k$ symbols that are independent with each other to represent the information. And other $n-k$ symbols are redundant ones that assist to complete the parity check. Thus, its code rate is defined as ${R}_{0}=k/n$. The code rate determines the correction capability, which means the maximum error rate that the LDPC code can correct. It has been shown that a given LDPC code with code rate *R* only can correct the error in a certain range^16,17^. Therefore, multiple LDPC codes are needed to cover a wide range of error rate. However, this solution may be impractical in the hardware implementation of a QKD system, because it occupies memory space to store copies of the LDPC codes. Thus, an ideal solution would be using a single LDPC code whose code rate can be adjusted in order to adapt to a wide range of error rate. This solution is called rate-adaptive information reconciliation, in which the adaptable code rate is realized by the techniques of puncturing and shortening^19,20,24^.

Puncturing deletes *p* symbols from the code words, and thus the LDPC code becomes $C(n-p,k)$^21,22^. The code rate is increased to be $R(p)=k/(n-p)$. The puncturing technique can be used in the information reconciliation to adapt the code rate as follows. The bit string *X* with length (i.e. $n-p$) is hold by Alice. According to the error rate, Alice and Bob determine the number of puncturing symbols, the value of $p$. Then Alice randomly fills $p$ bits into her bit string $X$, constructing the code word with length $n$. The syndrome of the constructed code word is calculated by Alice and sent to Bob to help him correct his bit string. At Bob side, Bob also randomly fills $p$ bits into his bit string Y and operates the decoding procedure. Thus, if the new code rate under puncturing technique, $R(p)$, can adapt to the error rate, Bob is able to correct the mismatch in his bit string with a high success rate. For a time-varying channel, the value of $p$ should be changed according to the different error rate over time. Thus, the length of Alice’s and Bob’s bit strings ($n-p$) also needs to be adapted to the changed value of $p$. This is inconvenient in practice.

In contrary to puncturing that increases the code rate via reducing the redundant bits, the technique of shortening decreases the code rate by increasing the ratio of redundant bits in a code word^23^. Specifically, shortening means that Alice tells the Bob the values of $s$ symbols in the $k$ symbols, which reduces unknown information. Thus, the code $C(n,k)$ turns to be $C(n-s,k-s)$, and the code rate is decreased to be $R(s)=(k-s)/(n-s)$. During the information reconciliation, Alice and Bob share the values of the shortened bits and their positions. Then Alice constructs a code word with length $n$ that contains $s$ bits of the shortened ones and $n-s$ bits of raw key. At Bob side, he also builds a string with $n-s$ bits of the raw key and the *s*-bit shortened part. Shortening indeed also changes the code rate by adjusting the amount of shortened bits. However, there is an issue similar to the puncturing technique. Regarding a time-varying channel, the value of *s* also has to be changed over time due to the variable error rate, which causes the same problem of modifying the bit length of Alice’s and Bob’s strings.

To overcome the problem of varying string length mentioned above but also take the advantage of the adaptive rate, we can combine the techniques of puncturing and shortening. The total symbols of puncturing and shortening are set to be $d=p+s$, which is a fixed value. Thus, the length of Alice’s and Bob’s bit strings are also fixed to be $m=n-d$. To modify the code rate to adapt to different error rates, the values of $p$ and $s$ are flexible to be changed. Since the puncturing technique increases the code rate but the shortening technique decreases the code rate, a proper code rate can be achieved by balancing the values of $p$ and $s$. The range of code rate that can be covered by the rate-adaptive LDPC code is

1 $${R}_{min}=\frac{k-d}{n-d}\le R\le \frac{k}{n-d}={R}_{max}\mathrm{}.$$

Therefore, the rate-adaptive reconciliation protocol can use one LDPC code to adapt to a wide range of code rate with help of puncturing and shortening.

### The design of the proposed protocol

The blind reconciliation protocol inherits the core idea of rate-adaptive information reconciliation. Instead of deciding the values of $p$ and $s$ at the beginning of the protocol when the error rate is estimated, the blind reconciliation starts without error estimation and thus does not fix the values of $p$ and $s$^25–27^. At the beginning, all $d$ bits are regarded as punctured bits. If Bob is not able to correct the error bits according to the syndrome that Alice sends to him, a fixed amount of bits will be revealed as shortened bits in the next round. That is, ${\Delta }_{i}=\Delta ,\,i\in [1,t]$. Here, Δ_*i*_ is the number of the shortened bits revealed in the *i*th round, and it is also named as the step size in the *i*th round. Thus, in each round, Bob gains more information than last round to correct the errors. This protocol can achieve relative high reconciliation efficiency with the cost of operation time due to multiple communication rounds.

To keep the advantage of high reconciliation efficiency and also speed up its operation, we propose a blind reconciliation protocol with variable step sizes as follows. In the protocol, Alice and Bob assume that the error rate is minimum at the beginning, and Alice only sends syndrome to Bob (Set $p=d$, $s=0$). That is, Bob tries to correct the error bits with minimal help from Alice. The increased number of rounds indicates that the error rate is higher than that Bob can correct and more help from Alice (i.e. the information of the shortened bits) is needed. Thus, to provide more help, instead of revealing a fixed number of shortened bits in each round, Alice can reveal an increased amount of shortened bits ${\Delta }_{i}={\Delta }_{i-1}+\delta$ ($\delta \in {\mathbb{R}}$) to Bob. It is notable that the amount of shortened bits is related to the number of rounds. When the number of round *i* is larger, the amount of shortened bits revealed in this round also becomes greater. In this way, Bob can receive more help from Alice, thereby accelerating the speed of Bob’s error correction. Thus, variable step sizes in the reconciliation protocol mean that revealed shortened bits ${\Delta }_{i}={\Delta }_{i-1}+\delta$ in the *i*th round is more than ${\Delta }_{i-1}$ in the $i-1$ th round.

The specific procedure of the blind reconciliation protocol with variable step sizes is as follows.

Step 1: Preparation. Assume $C(n,k)$ is a LDPC code. Alice/Bob prepares a bit string $X$/$Y$ with length $m=n-d$ from the raw key, which will be reconciled during this protocol. Set $p=d$, $s=0$ and ${\Delta }_{0}=0$ as initialization.

Step 2: Encoding. Alice constructs an $n$-bit code word *X*′ that consists of $m$-bit raw key and *d*-bit random symbols as punctured bits. Alice then calculates the syndrome of *X*′ and sends it to Bob. Also, Alice informs Bob the positions of the punctured bits but not their values.

Step 3: Error correction. Bob builds another $n$-bit string *Y*′ that consists of *m*-bit raw key at his side, $s$-bit shortened symbols, and $p$-bit puncturing symbols. Then Bob runs the error correction algorithm. If the syndrome calculated from the corrected string matches to that sent by Alice, Bob successfully recovers $X$ and the protocol stops. Otherwise, goes to Step 4.

Step 4: Information disclosure. If $s=d$, the protocol fails. Otherwise, Alice discloses ${\Delta }_{i}={\Delta }_{i-1}+\delta$ symbols to Bob, and the total shortened bits are $s=s+{\Delta }_{i}$. Then turn back to Step 3 for the *i*th iteration of information reconciliation.

In this protocol, assume the maximum iterations is $t$ and the step size in *i*th iteration is Δ_*i*_, then ${\sum }_{i=1}^{t}\,{\Delta }_{i}=s$.

In short, there are two main advantages in the proposed protocol. During the first few rounds, Alice reveals less shortened bits to Bob than the original blind reconciliation protocol. If Bob still can recover the correct code word, the reconciliation efficiency of the improved protocol with variable step sizes is better than the original one because of less disclosed information. If the error rate is relative high, Bob has to run more rounds of reconciliation with more help from Alice. In the improved protocol, the shortened bits revealed in each round are increased gradually, which can provide more information to Bob for his reconciliation. Thus, under the situation of high error rate, less rounds of communication are needed for the improved protocol with variable step sizes than that of the original one, which saves the operation time. The simulation results in the next section also verify these advantages.

It is noted that although we only use linear function to increase the step sizes gradually in this paper, the change pattern of the step size is not restricted. For instance, exponential and logarithmic functions can also be used according to the error rate.

## Protocol Simulation Results

To show the improved performance of the blind reconciliation protocol with variable step sizes, we compare the reconciliation efficiency and the number of iterations between the proposed protocol and the original blind reconciliation protocol in the simulation. In the experiments, 64800-bit LDPC codes with code rate ${R}_{0}=0.8,0.6,0.5$ are chosen to cover the error range of [$1 \% ,10 \%$]. 10% of code bits are used for adapting the code rate. That it, $d=6480$. Regarding the original blind reconciliation protocol, we set the maximum number of iterations $N=3,6,10$, which means, $\Delta =2160,1080,648$ shortened bits are revealed in every iteration, respectively. Under the same LDPC code setting, we simulate two types of the improved blind reconciliation protocol with variable step sizes. In the first case, Alice reveals ${\Delta }_{i}=648\times i$ shortened bits in *i*th iteration, and in the second case, Alice reveals ${\Delta }_{1}=590$ and ${\Delta }_{i}=589\times (i-1)$ shortened bits in *i*th iteration. As an important parameter, the reconciliation efficiency *f* is calculated as follows in the simulation^26^.

2 $$f=\frac{1-R}{h(\varepsilon )}=\frac{1-\frac{k-s}{n-p-s}}{h(\varepsilon )}=\frac{n-p-k}{(n-d)h(\varepsilon )},$$

where $\varepsilon$ is the error rate, and $h(\varepsilon )$ is the binary Shannon entropy. The best information reconciliation is to reach $f=1$. We run each reconciliation protocol for 30 times and take the averaged values of reconciliation efficiency and iteration number for each case.

**Figure 1:**
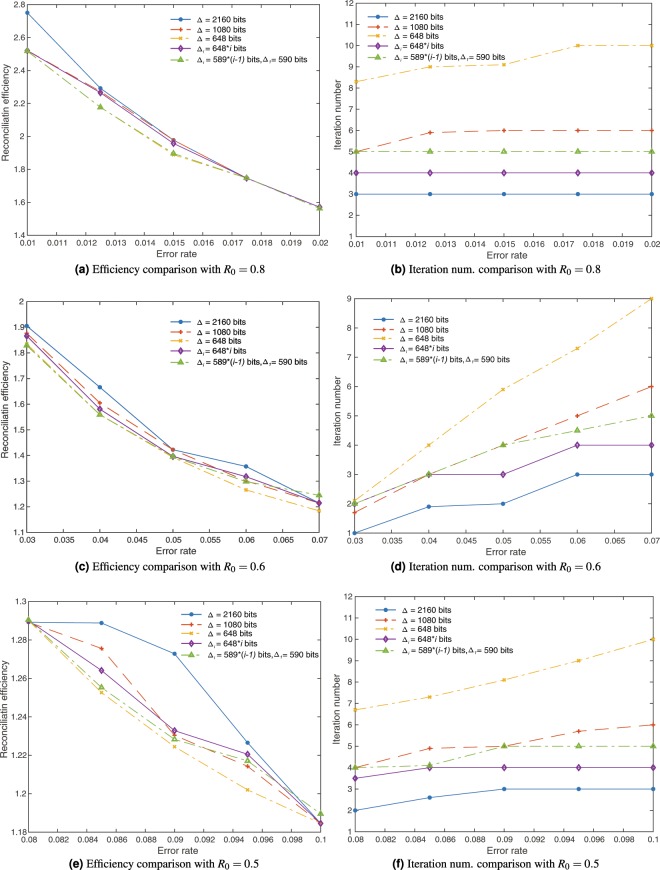
The simulated reconciliation efficiency and iteration number of the original blind reconciliation protocol (with $\Delta =2160$, $\Delta =1080$, $\Delta =648$ shortened bits revealed in every iteration) and the proposed blind reconciliation protocol (with ${\Delta }_{i}=648\times i$, ${\Delta }_{i}=589\times (i-1)$ shortened bits revealed in every iteration). To cover the error rate in the range [$\mathrm{1 \% }$, $\mathrm{10 \% }$], we simulate (**a**) the efficiency and (**b**) iteration number of the LDPC code with ${R}_{0}=0.8$, (**c**) the efficiency and (**d**) iteration number of the LDPC code with ${R}_{0}=0.6$, and (**e**) the efficiency and (**f**) iteration number of the LDPC code with ${R}_{0}=0.5$.

The simulation results are shown in Fig. 1. We can see that for the relative low error rates in Fig. 1(a,c,e), the improved blind reconciliation protocol with variable step sizes ${\Delta }_{i}=648\times i$ and ${\Delta }_{i}=589\times (i-1)$ can achieve a better reconciliation efficiency than the original one with $\Delta =1080$ and $\Delta =2160$ bits revealed in every iteration. The reconciliation efficiency of the protocol with the variable step sizes ${\Delta }_{i}=589\times (i-1)$ is close to the original one with small step of $\Delta =648$ bits revealed in every iteration. Thus, the better reconciliation efficiency achieved by the improved protocol discloses less information to the public, which helps Alice and Bob preserve more secret information. When the error rate is relative high in Fig. 1(a,c,e), the efficiency of the proposed blind reconciliation protocol may be slightly worse than that of the original protocol. However, in this range of high error rate, as shown in Fig. 1(b,d,f), the improved protocol with ${\Delta }_{i}=648\times i$ or ${\Delta }_{i}=589\times (i-1)$ operates less iterations than the original one with steps $\Delta =1080$ and $\Delta =648$ and also achieves similar reconciliation efficiency. This shows the improved protocol consumes much less time to process the phase of information reconciliation. This strategy accelerates the speed of post-processing especially for the cases of high error rate, which also saves hardware resources. In the application of the blind information reconciliation where the QBER is not known in advance, the advantages of our proposed protocol are obvious. Therefore, the improved blind reconciliation protocol with variable step sizes relieves the conflict of reconciliation efficiency and operation time by gradually revealing more number of shortened bits instead of a fixed number of shortened bits in each iteration.

## Secret Key Rate Analysis for QKD

To further shown the better reconciliation efficiency acheived by the improved blind reconciliation protocol can helps Alice and Bob generate higher secret key rate, we simulate the decoy-state BB84 QKD protocol with the reconciliation efficiency obtained by the simulation. According to the analysis of Gottesman-Lo-Lütkenhaus-Preskill (GLLP)^29^, the secret key rate of QKD with the weak coherent source can be written as

3 $$R\ge q\{\,-\,{Q}_{\mu }{H}_{2}({E}_{\mu })f({E}_{\mu })+{P}_{1}^{\mu }{Y}_{1}^{\mu }[1-{H}_{2}({e}_{1}^{\mu })]\},$$

where $q=1/2$ for BB84 protocol, *μ* is the intensity of a signal state, ${Q}_{\mu }$/${E}_{\mu }$ is the total gain/error rate of the signal state, ${Y}_{1}^{\mu }$ and ${e}_{1}^{\mu }$ are the yield and error rate of single-photon pulses in the signal states, ${P}_{1}^{\mu }$ is the probability of single-photon pulses in the signal states, $f(x)$ is the reconciliation efficiency, and ${H}_{2}(x)=-\,x\,{\log }_{2}\,(x)-(1-x)\,{\log }_{2}\,(1-x)$ is the binary Shannon information entropy. In the decoy-state protocol, ${Y}_{1}^{\mu }$ and ${e}_{1}^{\mu }$ are estimated as follows^30^.

4 $$\begin{array}{rcl}{Y}_{1}^{L} & = & \frac{\mu }{\mu \nu -{\nu }^{2}}({Q}_{\nu }{e}^{\nu }-{Q}_{\mu }{e}^{\mu }\frac{{\nu }^{2}}{{\mu }^{2}}-\frac{{\mu }^{2}-{\nu }^{2}}{{\mu }^{2}}{Y}_{0}),\\ {e}_{1}^{U} & = & \frac{{E}_{\nu }{Q}_{\nu }{e}^{\nu }-{e}_{0}{Y}_{0}}{{Y}_{1}^{L}\nu }.\end{array}$$

Here $\nu$ is the intensity of a decoy state, ${Q}_{\nu }$/${E}_{\nu }$ is the total gain/error rate of the decoy state, ${Y}_{0}$ is the dark count rate of a single-photon detector, and ${e}_{0}$ is the error rate of the background noise.

In order to show the effect of the improved efficiency provided by the modified blind reconciliation protocol on a QKD system, we simulate the final secret key rate of a decoy-state BB84 QKD system with the typical reconciliation efficiency obtained from the blind reconciliation protocol with shortened bits ${\Delta }_{1}=590$ and ${\Delta }_{i}=589\times (i-1)$. As a comparison, we also show the secret key rate with the reconciliation efficiency of the original blind reconciliation protocol with $\Delta =1080$ shortened bits revealed in each iteration. In the simulation, we assume $\mu =0.6$, $\nu =0.2$, and ${e}_{0}=0.5$. All the detection parameters are taken from the Gobby-Yuan-Shields (GYS) experiment^31^. The dark count rate ${Y}_{0}=1.7\times {10}^{-6}$, the transmittance in Bob’s device ${\eta }_{Bob}=4.5 \%$, and the misalignment error rate ${e}_{detector}=\mathrm{3.3 \% }$.

**Figure 2:**
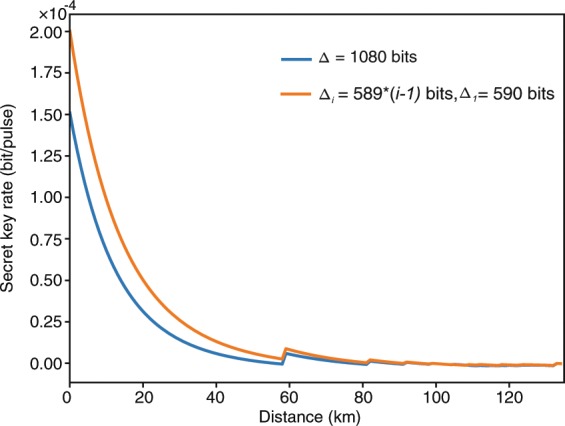
The simulated secret key rates of a decoy-state BB84 QKD system with the reconciliation efficiency obtained from the original blind reconciliation protocol ($\Delta =1080$) and the blind reconciliation protocol with variable step sizes (${\Delta }_{1}=590$ and ${\Delta }_{i}=589\times (i-1)$), respectively. The detection parameters are taken from the GYS experiment. The dark count rate ${Y}_{0}=1.7\times {10}^{-6}$, the transmittance in Bob’s device ${\eta }_{Bob}=\mathrm{4.5 \% }$, and the misalignment error rate ${e}_{detector}=\mathrm{3.3 \% }$.

The simulation results are shown in Fig. 2. From Fig. 2 we can see that the secret key rate with the reconciliation efficiency obtained from the reconciliation protocol with variable step sizes is higher than that with reconciliation efficiency got from the original blind reconciliation protocol. This is because the modified protocol with variable step sizes can achieve a higher reconciliation efficiency than the original one. The fluctuation points are the places where the reconciliation efficiency changed due to our simulation data of different error rates. The simulation results prove that the modified blind reconciliation protocol contributes to a higher secret key rate for the decoy-state BB84 QKD system than the original blind reconciliation protocol.

## Discussion and Conclusions

Information reconciliation is an important step in the post-processing of QKD. The information reconciliation protocol based on LDPC codes is becoming popular because of its strong capability of error correction. As required by practice, blind reconciliation protocol can correct the error without estimating the QBER but still obtain good reconciliation efficiency. However, the blind reconciliation protocol runs multiple rounds of communication between Alice and Bob to correct all errors, which consumes large amount of communication resources. Thus, reconciliation efficiency is a key parameter in QKD, and the speed of reconciliation is the bottleneck of the system’s repetition rate. In this work, we propose an improved blind reconciliation protocol with variable step sizes that reveals more shortened bits than that in the last round, increasing the information of the code word to help Bob correct his errors. The major modification of the improved reconciliation protocol is to set the number of shortened bits Δ_*i*_ disclose to Bob in the *i*th iteration is related to *i* instead of a fixed number as in the original protocol. This indicates the idea of variable step sizes.

The variable step sizes relieve the conflict of the reconciliation efficiency and the processing time. As shown by the analysis and simulation results in above sections, in the range of relative low error rate for a LDPC code, the improved blind reconciliation protocol discloses less shortened bits in the first several iterations, thus reaching better reconciliation efficiency than the original blind reconciliation protocol. In the range of relative high error rate, the proposed blind reconciliation protocol with variable step sizes reveals the shortened bits within less iterations than the original one to help Bob reconcile his bit strings, which takes less operation time and accelerates the speed of post-processing. In the application of the blind information reconciliation where the QBER is not known in advance, the advantages of our proposed protocol are obvious. We also show that the modified blind reconciliation protocol can provide a better reconciliation efficiency in QKD system, which enhances the final secret key rate of a decoy-state BB84 QKD system. To further achieve a even better reconciliation efficiency for the improved protocol, a protocol that can choose the size of step (Δ_*i*_) according to the error correction conditions of previous rounds in a more sophisticated way may be needed. This optimized protocol can be our future work. Moreover, the blind information reconciliation protocol with variable sizes may combine with the methodology of symmetric blind information reconciliation proposed in ref. ^28^ that improves the reconciliation efficiency by disclosing the positions of additional shortened bits decidedly indicated by unsuccessful belief propagation decoding algorithm. The reconciliation efficiency may be further improved by leveraging these two ways of the information leakage reduction. The investigation of this methodological combination can also be an interesting future work.
